# Kinetic Analysis of 4-Nitrophenol Reduction by “Water-Soluble” Palladium Nanoparticles

**DOI:** 10.3390/nano10061169

**Published:** 2020-06-15

**Authors:** Anas Iben Ayad, Denis Luart, Aissa Ould Dris, Erwann Guénin

**Affiliations:** 1 TIMR (Integrated Transformations of Renewable Matter), ESCOM, Centre de Recherche Royallieu, Université de Technologie de Compiègne, CS 60319, CEDEX, 60203 Compiègne, France; anas.iben-ayad@utc.fr (A.I.A.); aissa.ould-dris@utc.fr (A.O.D.); 2 Laboratoire TIMR, EA4297, ESCOM—École Supérieure de Chimie Organique et Minérale, 1 Allée du Réseau Buckmaster, 60200 Compiègne, France; d.luart@escom.fr

**Keywords:** Palladium nanoparticles, 4-nitrophenol reduction, Langmuir–Hinshelwood Model, kinetics, thermodynamics

## Abstract

The most important model catalytic reaction to test the catalytic activity of metal nanoparticles is the reduction of 4-nitrophenol to 4-aminophenol by sodium borohydride as it can be precisely monitored by UV–vis spectroscopy with high accuracy. This work presents the catalytic reduction of 4-nitrophenol (4-Nip) to 4-aminophenol (4-Amp) in the presence of Pd nanoparticles and sodium borohydride as reductants in water. We first evaluate the kinetics using classical pseudo first-order kinetics. We report the effects of different initial 4-Nip and NaBH_4_ concentrations, reaction temperatures, and mass of Pd nanoparticles used for catalytic reduction. The thermodynamic parameters (activation energy, enthalpy, and entropy) were also determined. Results show that the kinetics are highly dependent on the reactant ratio and that pseudo first-order simplification is not always fit to describe the kinetics of the reaction. Assuming that all steps of this reaction proceed only on the surface of Pd nanoparticles, we applied a Langmuir−Hinshelwood model to describe the kinetics of the reaction. Experimental data of the decay rate of 4-nitrophenol were successfully fitted to the theoretical values obtained from the Langmuir–Hinshelwood model and all thermodynamic parameters, the true rate constant *k*, as well as the adsorption constants of 4-Nip, and BH_4_^−^ (*K*_4-*Nip*_ and *K*_*BH*4−_) were determined for each temperature.

## 1. Introduction

The world of nanoscience involving metallic nanoparticles (mNPs) has been the subject of numerous scientific and technological research studies during recent years. Thus far, the art of synthesizing mNPs and nanoalloys in a large range with full control of different shapes and sizes has resulted in widespread applications in engineering, electronics, and catalysis [1]. mNPs such as palladium, platinum, and gold have already shown their potential in catalysis of several specific reactions, such as hydrogenation, water splitting, and cross-coupling reactions [2]. For example, bulk inert metals such as gold may become active in catalysis when divided into nanometer dimensions [3,4]. The redox potential value of metallic nanoparticles becomes progressively negative depending on their Fermi level that is related to their size [5,6,7]. 

4-Nitrophenol is a significant side product obtained from pharmaceutical industries, synthetic dyes, herbicides, and pesticides. Additionally, it is known as a hazardous product, and its removal from the environment is an important goal because of the negative effect on human and animal blood as well as several organs [8,9,10]. Therefore, its catalytic reduction by several mNPs has been a continuous subject of research for several years. The reduction of 4-nitrophenol by sodium borohydride is a model reaction to evaluate the catalytic activity of mNPs and to study the reaction kinetics. The main reasons for the use of this reaction as a model are that the reaction is well-controlled in the presence of the mNPs without by-product formation, and that the kinetics analysis of the reaction rate as a function of temperature is easily monitored by UV–vis spectroscopy. The presence of isosbestic points in the UV−vis absorption spectra at different times provides clear proof that 4-nitrophenol is completely reduced to 4-aminophenol with no side products generated. In addition, this reaction proceeds under mild conditions (at room temperature in water as a solvent). Thus, this model reaction has become a benchmark reaction of mNPs (see Table S1 in the Electronic Supplementary Information of the review of P. Hervés et al. [11]); the fundamental (kinetics) catalytic model reaction for mNPs has been described by several authors [7,12,13]. Almost all authors consider that the catalytic reduction of 4-nitrophenol (4-Nip) takes place on the surface of the mNPs [8,14], of which a prerequisite is that all reactants, 4-Nip, and borohydride ion must be adsorbed on the surface of mNPs to react. The apparent rate constant *k_app_* is determined in terms of pseudo first-order kinetics. 

In this work, we propose to study the catalytic reduction of 4-nitrophenol (4-Nip) to 4-aminophenol (4-Amp) in the presence of Pd NPs and sodium borohydride as a reductant in water and to study the kinetics of the reaction. These Pd NPs are obtained in a simple and eco-friendly manner and have already shown high efficiency for several organic reactions in aqueous media [15]. Therefore, understanding their kinetics in a well-defined model such as 4-Nip reduction is of interest.

## 2. Materials and Methods 

### 2.1. Chemicals

Na_2_PdCl_4_, sodium ascorbate, and NaBH_4_ were purchased from Sigma–Aldrich (Saint-Quentin Fallavier, France), while 4-nitrophenol was acquired from Acros Organics - Fisher Scientific (Illkirch, France). All materials and solvents were used as received without further purification. Nanopure water (18 MΩ cm^−1^), obtained from a Millipore Gradiant Elix-3A10 system (Millipore SAS, Molsheim, France), was used for all experiments. Synthesis and characterization of the Pd NPs as well as their bisphosphonic acid ligand were performed using the protocols described in our previous work [15].

### 2.2. Dynamic Light Scattering (DLS) Analysis

The average hydrodynamic radius of Pd nanoparticles was observed in water at pH = 7 by a DLS (Dynamic Light Scattering) Litesizer 500 Anton Paar particle size analyzer (Anton Paar France, Courtabeouf, France). The measurements were performed in quartz cuvettes at a temperature 293.15 K, laser wavelength 658 nm (single-frequency laser diode), detection angle 175°, medium viscosity 0.8903 mPa·s, and medium refractive index 1.3303.

### 2.3. Spectrophotometer (UV–vis) Analysis

The catalytic reduction of 4-nitrophenol was monitored by a Perkin–Elmer Lambda-2 UV–vis Spectrophotometer (PerkinElmer France, Villebon-sur-Yvette, France) employing a 1 cm path length quartz cuvette (Hellma). The experimental error of the wavelength values was estimated to be 1 nm.

### 2.4. Catalytic Reduction of 4-Nitrophenol

The catalytic reduction of 4-nitrophenol (4-Nip) to 4-aminophenol (4-Amp) in the presence of NPs and sodium borohydride as a reductant was previously described in the literature [16,17,18]. Briefly, the UV absorption measurements were performed in a 3 mL quartz cell. The solutions of 4-Nip and NaBH_4_ were freshly prepared before each measurement, without purging with N_2_ [16]. In a typical experiment, 30 μL of 4-Nip (7.5 × 10^−3^ M) solution was added to the reaction medium (2.8 mL of water) with 200 μL of sodium borohydride solution (0.169 M); the ratio of sodium borohydride to 4-Nip concentration in the medium was equal to 150. Under these conditions, all 4-Nip was transformed into 4-nitrophenolate, which was detected at *λ* = 400 nm. Then, 20 μL of NP Pd solution (2.8 × 10^−5^ M) was added to the solution. Immediately after the addition of Pd NPs, time-dependent absorption spectra were recorded at 6 s intervals for different temperatures.

## 3. Kinetics Analysis

The kinetic data were examined first in terms of pseudo first-order kinetics and then fitted to a Langmuir–Hinshelwood model. There are two types of catalytic mechanisms of 4-Nip reduction. The first one is surface-mediated hydrogen transfer, described by several authors and especially the Ballauff group [8,12,13,16,19], and the second one is surface-mediated electron transfer [20,21].

We assume that both catalytic mechanisms are involved in this reaction. Both reactants, 4-Nip and borohydride ions, must absorb onto the surface of mNPs before the reduction of 4-Nip. The reaction goes through two stages: the first stage includes two steps, the equilibrated adsorption of borohydride ions and production of the hydrogen radical by electron transfer [22,23]. 

**Figure 1:**
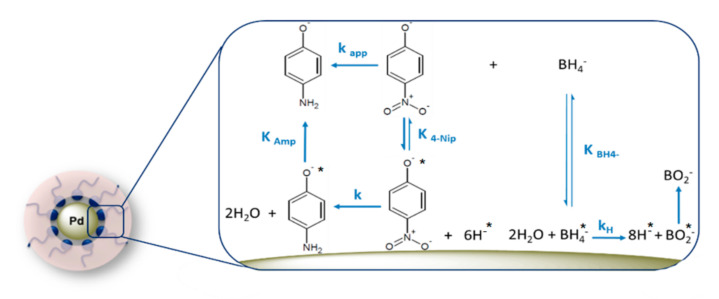
Schematic representation of the Langmuir–Hinshelwood mechanism of 4-Nip reduction on the surface of Pd nanoparticles.

The second stage includes three steps, the equilibrated adsorption of 4-Nitrophenolate, the addition of hydrogen species to the adsorbed molecule to form the 4-aminophenol via the formation of the 4-hydroxylaminophenol intermediate [16,24], and the removal of two water molecules from the nitro group, and finally the detachment of the 4-aminophenol molecule from the nanoparticle surface to provide place for another catalytic cycle. Figure 1 displays the essential process of 4-Nip reduction on the surface of Pd nanoparticles based on the Langmuir–Hinshelwood mechanism.

The adsorption of 4-Nip on the surface of the Pd NPs is reversible and is modeled by a Langmuir isotherm [25]. Previous kinetic studies indicated that the reduction of the 4-Nitrophenol molecule adsorbed on the surface, including the surface hydrogen species, is the rate-determining step [13]. Some authors reported that the interaction of borohydride ions with metallic surfaces is a complex mechanism that constitutes several stages [26,27]. Moreover, the production of a surface-hydrogen species by the adsorbed borohydride ion and the final detachment of 4-aminophenol molecule are fast and irreversible; for that reason, these steps do not enter into the Langmuir–Hinshelwood equations [12]. The Langmuir–Hinshelwood equation can be defined as follows:

(1) d4-Nipdt=−kSθ4-Nip⋅θBH4−=−kapp⋅4-Nip

where *k* is the rate constant, *S* is the total surface of all Pd nanoparticles, accurately proportional to the apparent kinetic constant *k_app_*, and *θ_Nip_* and *θ_BH4_* indicate the surface coverage values on the surface of 4-*Nip* and *BH*_4_^−^, which can be expressed by a Langmuir-Freundlich isotherm:

θ4-Nip=(K4-Nip⋅4-Nip)n(1+(K4-Nip⋅4-Nip)n+(KBH4−⋅BH4−)m

θBH4−=(KBH4−⋅BH4−)m(1+(K4-Nip⋅4-Nip)n+(KBH4−⋅BH4−)m

where *K*_4-*Nip*_, *K*_*BH*4_^−^, [4-*Nip*], and [*BH*_4_^−^] are the adsorption constants and the concentrations of 4-*Nip* and *BH*_4_^−^, respectively. The exponent n and m are related to the adsorption and desorption equilibrium of 4-*Nip* and *BH*_4_^−^ molecules, respectively, and they indicate the heterogeneity of the sorbent. The heterogeneity parameters *n* and *m* are both equal to 1 according to the classical Langmuir isotherm model [28].

This basic model considers that the surface of the metal catalyst is homogeneous in terms of adsorption energy, which means that the adsorption energy is the same for all sites. Thus, the maximal adsorption capacity is full monolayer coverage, without interaction between adsorbed species. The model of the Langmuir isotherm equation can be defined as follows [28]:

qe=qm⋅(KL⋅Ce)1+KL⋅Ce

where *q_e_* is the amount of adsorbate (4-Nip) adsorbed on metal nanoparticles, *q_m_* is the maximum saturation load, i.e., the mass of 4-Nip required per gram of metal nanoparticles to form a complete monolayer on the surface, *K_L_* is the Langmuir affinity constant related to the equilibrium constant or binding energy, *K_L_αe^−ΔH/RT^*, and *Ce* is the equilibrium concentration of 4-Nip in solution. The past finding of an exponent *n* = 1 by some authors for adsorption of 4-nitrophenol and 4-aminophenol may therefore point to the identical outcome [25,29].

Hence, Equation (1) can be rearranged and rewritten as:

d4-Nipdt=−k⋅S⋅K4-Nip ⋅4-Nip⋅KBH4−⋅BH4−(1+K4-Nip⋅4-Nip+KBH4−⋅BH4−)2

Thus, *k_app_* can follow as:

kapp=k⋅S⋅K4-Nip⋅KBH4−⋅BH4−(1+K4-Nip⋅4-Nip+KBH4−⋅BH4−)2

The true rate constant *k* is linked to the reaction of the adsorbed species *K*_4-*Nip*_ and *K*_*BH4*_ [8]; these constants can be obtained from a fit of the temporal decay of the concentration of 4-Nip to the series of experimental data (see the procedure in the Supporting Information).

In order to obtain the adsorption constants *K*_4-*Nip*_ and *K_BH_*_4^−^_ as a function of temperature, the enthalpies and entropy for the adsorption of 4-*Nip* and *BH*_4_^−^ can be obtained from the application of Van’t Hoff’s equation and by combining the Gibbs free energy equation, respectively.

Van’t Hoff’s equation:

d ln Kd1/T=−ΔHR

Combining the Gibbs free energy equation:

ln K=−ΔGRT=−ΔHRT+ΔSR

## 4. Results and Discussion

### 4.1. Catalytic Reduction of 4-Nitrophenol Using Pd NPs

**Figure 2:**
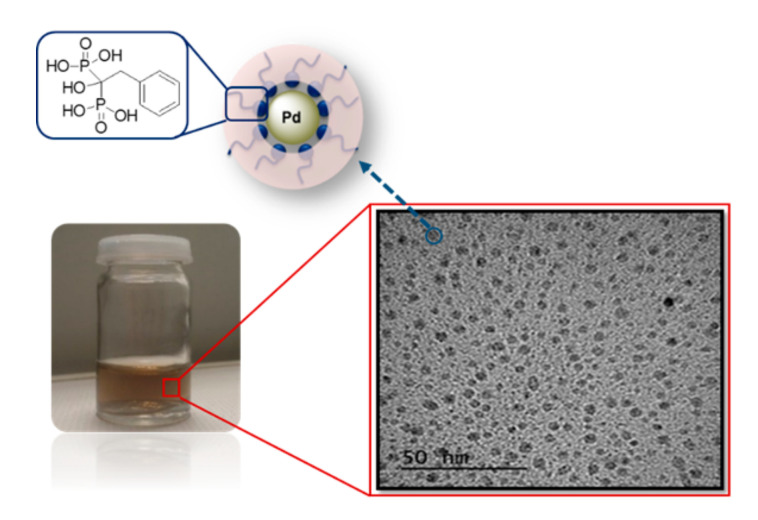
Structure of the Pd NPs used in the kinetic studies. Lower panel, right side: TEM micrograph of Pd-NPs solution (average diameter, 6 nm). The upper panel left side: Scheme of the Pd NPs consisting of spherical Pd with benzyl bisphosphonic acid as a ligand (HMBP).

Pd NPs have been shown to be excellent catalysts for different reactions performed in aqueous media, such as carbon–carbon cross-coupling, hydrogenation of alkenes, and oxidation of primary alcohols as shown in our previous work [15]. We used benzyl bisphosphonic acid as a ligand for the nanoparticles (see Figure 2). The median diameter size of the Pd nanoparticles obtained from the TEM micrographs was 6 nm. The specific surface area (*S*) was calculated based on the median radius [8] and was used in the determination of the kinetic data.

**Figure 3:**
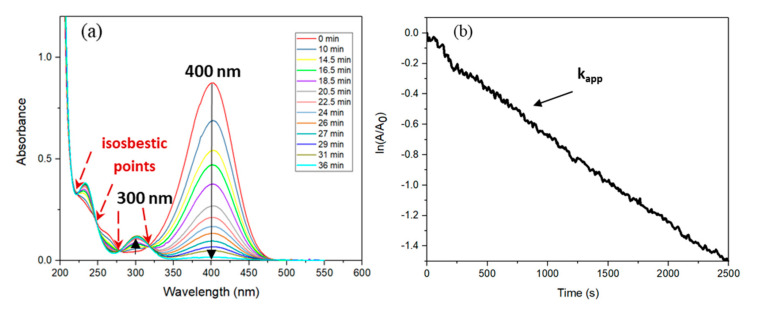
(**a**) Following UV–vis absorption spectra (6 s intervals) for the catalytic reduction of 4-Nip using Pd nanoparticles (pH = 10). (**b**) Absorption dependence of the time for the reduction of 4-Nip. Conditions: [4-Nip] = 7.38 × 10^−5^ M; [NaBH_4_] = 1.11 × 10^−^^2^ M (150 eq (equivalent)); [Pd] = 2.8 × 10^−5^ M (0.249 mol% Pd); *T* = 23.7 °C. (*k_app_* = 0.0006 s^−1^).

Figure 3a shows the results of UV–vis spectra for the catalytic reduction of 4-nitrophenolate ions. The absorption intensity of 4-nitrophenolate ions at 400 nm decreased and on the other hand, the absorption of 4-aminophenol at 300 nm appeared. The observation of four isosbestic points demonstrates that no side products were produced [30]. Figure 3b shows the ordinary absorption dependence over time for the reduction of 4-nitrophenolate ions at a wavelength of 400 nm. In our case, contrary to what was observed by Wunder and co-workers [12,13,16], no induction time was noticed. On the other hand, Menumerov showed that the induction period was linked to the level of dissolved oxygen contained in the medium [31]. He determined that the induction period ended once the level of dissolved oxygen fell below a critical value and that this level was dependent upon the metal nanoparticles used as a catalyst.

As can be seen, the reaction is stationary, and the linear fitting degree of ln(*A*/*A*_0_) versus time follows pseudo first-order kinetics. The apparent kinetic rate constant (*k_app_*) was determined from linear regression using the following equation:

ln(AA0)=ln(CC0)=kapp⋅t

where *A*_0_ is the initial absorbance, and *A* is the absorbance of 4-nitrophenolate ions at every time point. *C_0_* is the initial concentration, and *C* is the concentration of 4-nitrophenolate ions in the reaction medium. According to the slope, the apparent rate constant is equal to 0.0006 s^−1^. 

### 4.2. Influence of Temperature (Derivation of Thermodynamic Parameters)

**Figure 4:**
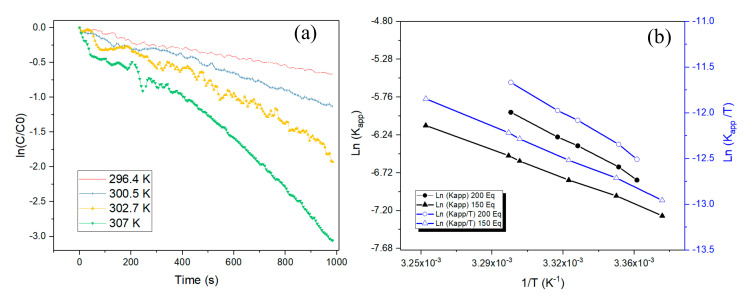
(**a**) ln (*C/C_0_*) versus time at different reaction temperatures, conditions of reaction 150 eq BH_4_^−^; (**b**) Arrhenius plots ln(*k_app_*) and Eyring plots ln(*k_app_/T*) versus 1/*T* for the Pd nanoparticles.

The activation energy and thermodynamic parameters of the activation enthalpy and entropy for the reduction of 4-Nitrophenol by Pd nanoparticles were determined by conducting the reaction at various temperatures. As can be seen from Figure 4a, the constant *k_app_* increases with increasing the temperature, which can be made clear by collision theory.

The movement of the 4-Nitrophenol molecule is considered to be vigorous, leading to a better chance for collision when we increase the temperature of the medium, which results in the rapid reaction rate [32]. The apparent activation energy (*Ea_app_*), pre-exponential factor *A*, enthalpy (ΔH^‡^), entropy (ΔS^‡^), and Gibbs energy of activation (ΔG^‡^) were calculated using the following equations:

Arrhenius equation:

lnkapp=lnA−EaappRT

Unimolecular reaction equation:

A=e1⋅kB⋅T°h⋅eΔS‡R

Eyring equation:

lnkappT=−ΔH‡R⋅1T+ΔS‡R+lnkBh

Gibbs equation:

$$\Delta G^{‡}=\Delta H^{‡}-T\times \Delta S^{‡}$$

where *R* is the ideal gas constant (*R* = 8.314 J·K^−1^·mol^−1^), *h* is Planck’s constant (*h* = 6.63 × 10^−34^ J·s), *k_B_* is the Boltzmann constant (1.38 × 10^−23^ J·K^−1^), T is the absolute temperature, and ΔH^‡^, ΔS^‡^, and ΔG^‡^ are the activation of enthalpy, entropy, and Gibbs energy, respectively.

As shown in Table 1, the apparent activation energy (*Ea_app_*) was 81.22 kJ·mol^−1^ and 112.77 kJ·mol^−1^ for 150 and 200 equivalents of NaBH_4,_ respectively, indicating that the apparent activation energy for the 4-Nip reduction was increased by increasing the NaBH_4_ equivalent. The *Ea* was affected by the concentration of sodium borohydride, which plays a critical role in the pH medium. The pH medium affects the overall rate constant and activation energy. In the case of 150 eq, the pH was 9.69, and in the case of 200 eq, the pH was 9.93. This slight change influenced the NaBH_4_ hydrolysis, which was faster, leading to higher formation of reactive Pd-H at the surface of NPs as described by Grzeschik and coworkers [33]. They found that the pH value has an influence on the kinetics of 4-Nip reduction and that by decreasing the initial pH value, the reaction becomes much faster. Consequently, by increasing the concentration of NaBH_4_ in the medium, we increased the hydrogen and hydride species formation in the medium, which led to the saturation of the surface of Pd NPs (Pd–H) followed by increased *Ea*. 

The *Ea* value was relatively similar for reactions over platinum and/or palladium nanocages (NCs). It was comparable to the values reported for the reduction of 4-Nip over Pt–Pd alloy NCs (109.6 kJ·mol^−1^) and Pd NCs (94.5 kJ·mol^−1^) [34].

According to the plot of ln (*k_app_/T*) versus 1/*T*, we found the values of ΔH^‡^ positive for both cases (150 eq and 200 eq), which means that the 4-nitrophenol reduction is endothermic [19,35] due to the fact that the product, 4-aminophenol, has greater enthalpy than the reactant, 4-nitrophenol. The value of ΔS^‡^ provides an indication of the molecularity of the rate-determining step, that is to say if the reactants are bound to each other or not. We noticed a negative value of ΔS^‡^ in the case of 150 eq, which signifies that entropy decreased as more ordered structures for the transition state were formed. This frequently indicates an associative mechanism of the adsorption of 4-nitrophenol as well as BH_4_^−^ on the surface of Pd nanoparticles to form a single activated complex [36,37]. In the case of 200 eq, we obtained a positive value, indicating an increase in achieving the transition state, which frequently signifies a dissociative mechanism in which the activated complex is loosely bound and about to dissociate. This change in ΔS‡ is due to the compensation effect between the transition state entropy and enthalpy that was observed by Corma et al. [38]. Generally, the compensation effect has been explained by many effects such as the relationship between the coverage of the surface and activation energy [39,40], entropy–enthalpy relationship [39,41], and many other effects that were further investigated by Bond et al. in their article review [39].

The Gibbs free energy of activation ΔG^‡^ was determined at 25 °C; this free-energy indicates the barrier to move from the 4-nitrophenol state to the activated state. We found almost the same values, 90.47 kJ·mol^−1^ and 89.56 kJ·mol^−1^, for 150 eq and 200 eq, respectively, which means that reduction of 4-nitrophenol to 4-aminophenol required the same energy. 

### 4.3. Effects of Surface Area and Concentration of Reagents 

Many authors have reported that the amount of nanocatalyst, the initial concentration of 4-nitrophenol, and NaBH_4_ are important factors that influence the catalytic reduction of 4-nitrophenol to 4-aminophenol by a metal nanoparticle [12,13,42,43]. To study the effect on the apparent rate constant (*k_app_*) at 25.5 °C, the experiments were carried out by varying a single factor of the initial amounts of the nanocatalyst, 4-nitrophenol, and NaBH_4_ concentration.

**Figure 5:**
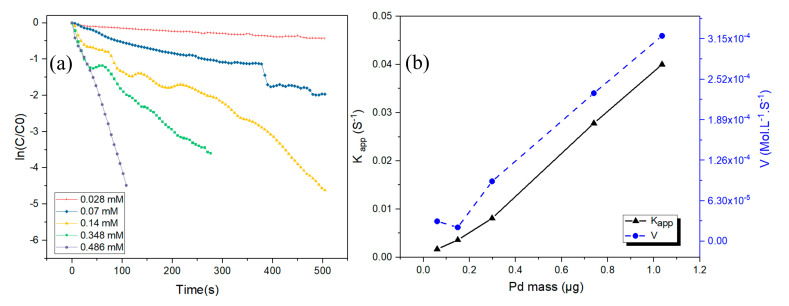
(**a**) ln (*C/C_0_*) versus time at different concentrations of Pd nanoparticles. (**b**) Plots of *k_app_* and initial velocity (rate) versus the amount of Pd nanocatalyst; conditions of the reaction at 150 eq NaBH_4_; [4-NIP] = 7.38 × 10^−5^ M; temperature 25.5 °C.

Furthermore, each number of active sites that exist at the surface of nanoparticles is proportional to the amount of Pd nanoparticles used. In this manner, as predicted, Figure 5b shows that the value of *k_app_* and the initial velocity increase with an increase in the amount of the nanocatalyst.

**Figure 6:**
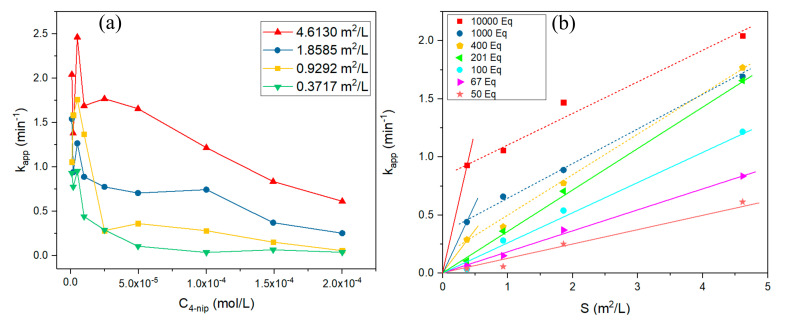
Plots of *k_app_* versus (**a**) the concentration of 4-nitrophenol at a constant temperature of 22.4 °C and concentration of NaBH_4_ of 1 × 10^−2^ M for four different surface areas of NPs Pd, 0.3717 m^2^/L, 0.9292 m^2^/L, 1.8585 m^2^/L, and 4.6130 m^2^/L. (**b**) Surface area of Pd NPs at a constant temperature of 22.4 °C for seven different equivalents of NaBH_4_ 10,000 eq, 1000 eq, 400 eq, 201 eq, 100 eq, 67 eq, and 50 eq. The straight lines illustrate the intrinsic kinetic area, and the dashed lines illustrate the area where diffusion limitations take place.

Figure 6a,b display the plots of *k_app_* at different initial 4-nitrophenol concentrations and the surface area, respectively. As we noticed from Figure 6a, the value of *k_app_* decreased in the case of increasing the initial 4-nitrophenol concentration. Up to certain concentrations and surface areas, the constant *k_app_* remained constant. The empirical model using classical methods of experimental kinetics is not valid since the constant *k_app_* is constant only for a well-defined area (from C4-nip ranging from 5 to 2 × 10^−4^ M), and the active surface (quantity of nano of Pd) of *k_app_* is related to the surface area of the Pd nanoparticles.

To describe the model, we must turn to the Langmuir Hinshelwood model, which assumes that the adsorption of molecules onto the nanoparticles surface is fast and reversible.

The co-adsorption of 4-Nip and NaBH_4_ is required, as we assumed that all steps of this reaction proceed only on the surface-active sites of Pd nanoparticles (Langmuir−Hinshelwood model). However, when the initial 4-nitrophenol concentration in the solution is high, the active sites are mostly occupied by 4-nitrophenol molecules and a minority by active hydrogen species, which result in a low constant of the velocity of reaction. On the other hand, as the surface area increases and the initial concentration of 4-nitrophenol remains constant or decreases, following an increase in the NaBH_4_ to 4-Nip ratio, fewer 4-nitrophenol molecules adsorb on the surface because the majority of active sites are occupied by active hydrogen species, so the velocity of the reaction increases. 

Figure 6b shows the plot of the apparent kinetic rate constants obtained experimentally relative to the normalized surface area of Pd nanoparticles for different concentration ratios of NaBH_4_ to 4-Nip. As can be seen, the constant *k_app_* initially increased linearly with increasing surface area at a constant temperature and concentration ratio of NaBH_4_ to 4-Nip, which emphasizes the important effect of the amount of potential active sites based on the total surface area accessible for the catalytic reduction of 4-nitrophenol. The rate constant *k*_1_ normalized to the surface area was determined graphically by the equation:

$$k_{app}=k_{1}\cdot S$$

In each graph, the linear fitting indicates the intrinsic kinetic region (straight lines); the rate constant *k*_1_ normalized to the surface area was determined for each concentration ratio of NaBH_4_ to 4-Nip where 10,000 eq, 1000 eq, 400 eq, 201 eq, 100 eq, 67 eq, and 50 eq showed values of 2.5, 1.18, 0.78, 0.36, 0.26, 0.18, and 0.13 min/m^2^ L, respectively. The rate constant *k*_1_ normalized to the surface area showed a decrease in value from 10,000 eq to 50 eq. This was expected as the minority of active sites were occupied by active hydrogen species, so the velocity of the reaction decreased. The dashed lines represent the area where diffusion control takes place [8].

The diffusion control is a significant factor that must be taken into consideration in the kinetic studies of a catalytic reaction. The reactions occur only on the surface-active sites of Pd nanoparticles according to the Langmuir−Hinshelwood model. Therefore, when the rate of the reaction is significantly faster than the diffusion of the 4-nitrophenol molecules, the reaction is controlled by diffusion, and the constant *k_app_* notably includes the rate of diffusion of the 4-nitrophenol molecules through the medium. On the other hand, when the diffusion rate is faster than the rate of the reaction, the reaction is kinetically controlled, and the constant *k_app_* only indicates the rate-determining step. 

The ratio of these two rates can be described by the second Damköhler (DaII) equation [44]:

$$Da\mathrm{II}=\frac{chemical\;reaction\;rate}{mass\;transfer\;rate}=\frac{k_{app}\;{\left[4-\mathrm{Nip}\right]}^{n-1}}{\beta \;\alpha }$$

where *k_app_* is the apparent rate constant, n is the order of the reaction (first-order for 4-Nip), *α* is the interfacial area, which is the volume-normalized area of the Pd nanoparticles [19], and *β* is the mass transport coefficient, defined as the ratio of *D*, the diffusion coefficient of 4-*Nip* (6.92 × 10^−10^ m^2^/s) [45], to *δ*, the characteristic diffusion length, which is determined by DLS as the hydrodynamic radius of our Pd nanoparticles. The summary of these parameters for a different amount of Pd nanoparticles and two equivalents of NaBH_4_ is presented in Table 2.

**Figure 7:**
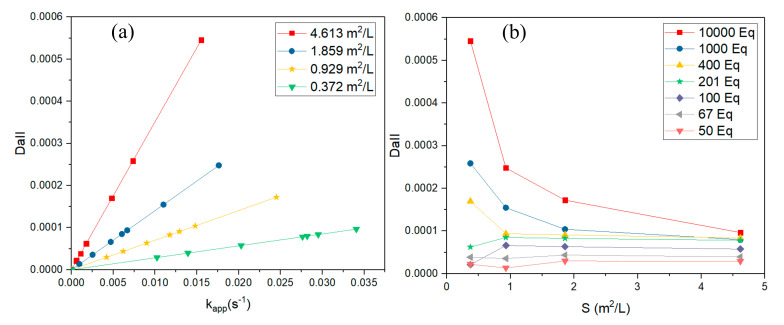
Plots of DaII versus different (**a**) apparent rate constants, *k_app_,* at a constant temperature of 22.4 °C and concentration of NaBH_4_ of 1 × 10^−2^ M at four different surface areas of Pd NPs, 0.3717 m^2^/L, 0.9292 m^2^/L, 1.8585 m^2^/L, and 4.6130 m^2^/L. (**b**) Surface area of NP Pd at a constant temperature of 22.4 °C for seven different equivalents of NaBH_4_: 10,000 eq, 1000 eq, 400 eq, 201 eq, 100 eq, 67 eq, and 50 eq.

Figure 7a,b display the plots of DaII at different *k_app_* and surface area values for different concentration ratios of NaBH_4_ to 4-Nip, respectively.

Figure 7a shows that the value of DaII increased in the case of the increase of the apparent rate constant *k_app_* at a constant surface area of Pd NPs. On the other hand, we noticed from Figure 7b that the value of the second Damköhler number decreased when we increased the surface area of Pd NPs for all cases until this number remained constant at a higher surface area. This result was expected since the initial concentration of NaBH_4_ was higher than that of 4-nitrophenol, and a lower surface area allows more active sites to be occupied by active hydrogen species.

The calculations of the second Damköhler number from 50 until 10,000 equivalents of NaBH_4_ showed values in the order of 10^−4^ or less. The diffusion control was negligible in all cases by reason that the values obtained were lower than 1 (DaII << 1), so the diffusion rate was much faster than the rate of reaction [8,46]. 

### 4.4. Influence of Reagent Concentration (Derivation of the Langmuir–Hinshelwood Parameters)

We investigated the fitting of the Langmuir–Hinshelwood model (Equation (1)) to experimental data determine by the temporal decay of the concentration of 4-Nip. Two different experiments were run at four different temperatures; by using Pd nanoparticle catalysts, the initial concentration of 4-Nip was set constant (7.38 × 10^−5^ M), and the initial concentration of BH_4_^−^ was varied from 150 to 200 eq.

**Figure 8:**
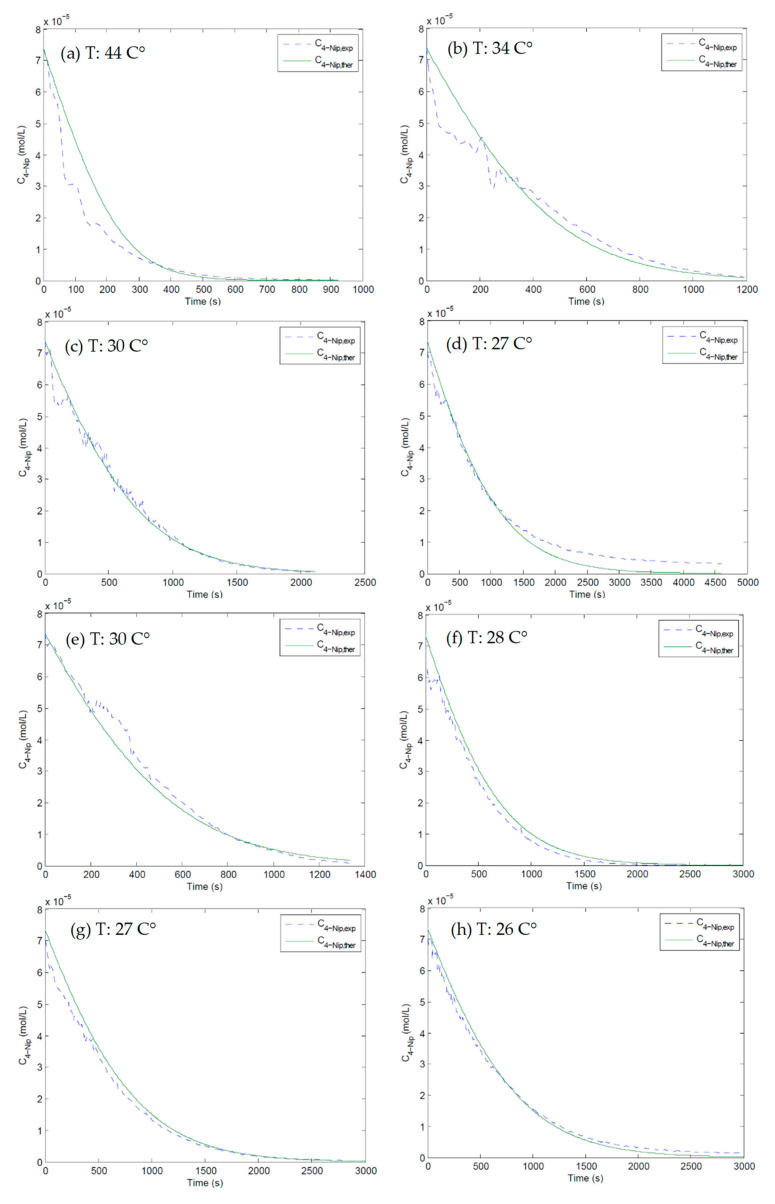
Plots of the concentration of 4-Nip as a function of time at different temperatures by using the Langmuir–Hinshelwood model. The solid green lines represent the respective fits to the kinetic model. The amount of BH_4_^−^ used was 150 eq and 200 eq for graphs (**a**–**d**) and (**e**–**h**), respectively.

Figure 8 displays the fits of Langmuir–Hinshelwood model to the experimental data at different temperatures. All curves were plot to a conversion of 60% to make sure of the significance of the comparison. The dashed lines are the experimental concentrations of 4-Nip, and the solid lines are the fits from the Langmuir–Hinshelwood model. As can be seen, C_4-Nip,ther_ deviates from C_4-Nip,exp_ at higher temperatures and longer reaction time, which is expected from inescapable side reaction of BH_4_^−^ hydrolysis [31,32], resulting in the shift of C_4-Nip,exp_ throughout the measurements.

**Figure 9:**
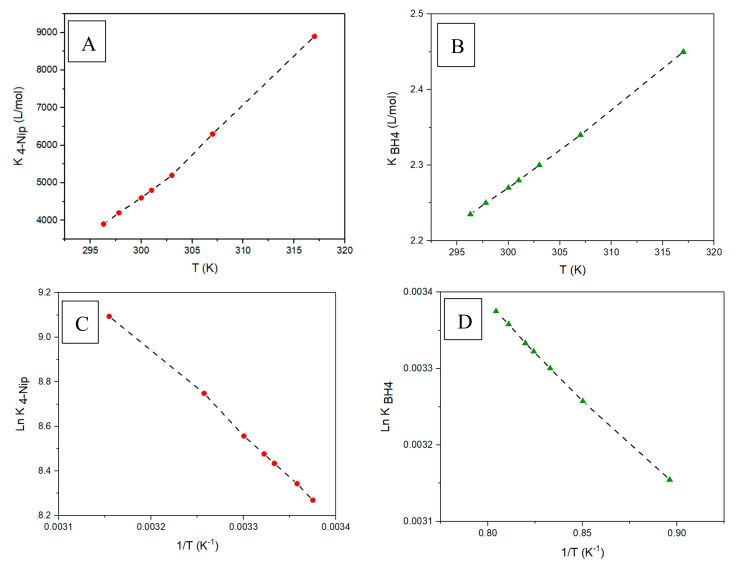
Plots of the adsorption constant *K*_4-*Nip*_ of 4-Nip (**A**) and the adsorption constant *K*_*BH*4_^−^ of BH_4_^−^; (**B**) versus temperature. The enthalpies and entropies of the adsorption process were calculated from ln(*K*_4-*Nip*_) (**C**) and ln(*K*_*BH*4_^−^) (**D**) based on the inverse of temperature using the van’t Hoff equation. All values are given in Table 4.

The corresponding fit parameters are presented in Table 3. These parameters were determined using a numerical solution of Equation (1) by a MatLab routine. Apparently, a single set of the rate constant *k* and the adsorption constants *K*_4-*Nip*_ and *K*_*BH*4−_ is able to represent the experimental data at a specific temperature. The value of the adsorption constant *K*_4-*Nip*_ is much higher than that of *K*_*BH*4−_ and increases with temperature, while K_BH4_ remained almost constant in the same temperature range (see Figure 9). This explains why the 4-nitrophenol molecules were more strongly adsorbed on the surface of the Pd nanoparticles than the borohydride ions. On the other hand, Guella and Liu showed that the interaction of borohydride ions with metal surfaces is a complex process that involves several steps [26,27].

**Figure 10:**
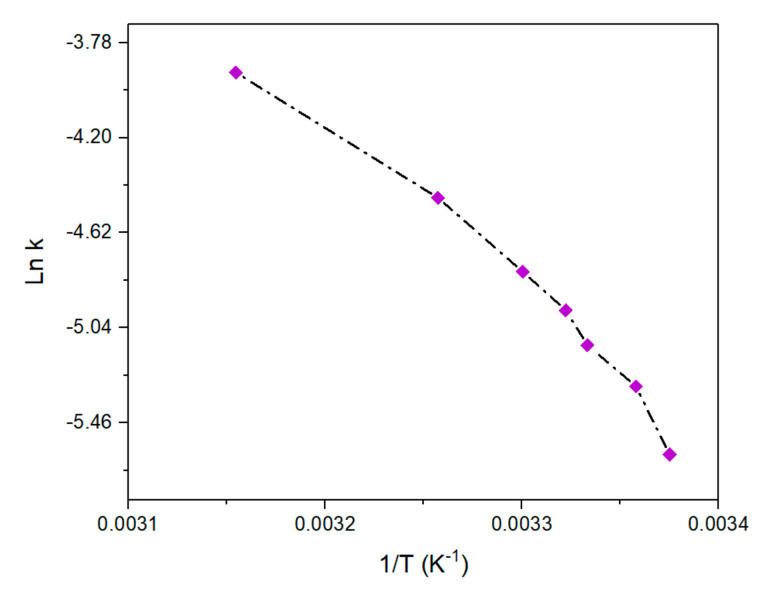
Arrhenius plots of ln k versus 1/*T* for the Pd nanoparticles at a constant concentration of 4-nitrophenol (7.38 × 10^−5^ M) and NaBH_4_ (150 eq). The rate constant *k* is obtained from fitting the experimental data with the Langmuir–Hinshelwood model (see Table 3).

Furthermore, the true rate constant k increased with an increase in the temperature, and the true activation energy *Ea* obtained from the Arrhenius plot was 61 kJ·mol^−1^ (Figure 10). This true activation energy is the same order of magnitude as that of apparent activation energy *Ea_app_* (81.22 kJ·mol^−1^) determined at 150 eq of *BH*_4_^−^ and is correlated with the reaction of the surface-bound species with adsorbed 4-nitrophenol. On the other hand, *Ea_app_* is just an apparent value as it contains the adsorption constants *K*_4-*Nip*_ and *K*_*BH*4_^−^, which depend on the temperature.

The values of thermodynamic parameters for the adsorption process of 4-Nip and BH_4_^−^ are given in Table 4. All enthalpy and entropy values of the adsorption of 4-Nip and BH_4_^−^ are positive. The adsorption entropy indicates that the release of water or other surface-bound species is the dominating process of adsorption [12]. These enthalpy and entropy adsorption values are in a similar range of what Wunder and coworkers found by using Gold NPs [12]. 

## 5. Conclusions

In summary, “water-soluble” Palladium Nanoparticles exhibit high catalytic performance for catalytic reduction of 4-nitrophenol by using sodium borohydride as the reducer in comparison to many various mNP catalysts reported in the literature (see Table 2 of the review [47]). All kinetics of the reduction process were studied by varying a single factor each time, i.e., the amount of Pd nanoparticles, initial 4-nitrophenol, and NaBH_4_ concentration under various experimental conditions. 

The evaluation of diffusion control was conducted by the calculation of the second Damköhler number. In this study, we found that the more we increased the concentration of NaBH_4_ in a small surface area of Pd nanoparticles, the more the second Damköhler number increased. However, under this condition, the catalytic systems were not controlled by the diffusion of reactants to the surface of the nanoparticles because the values obtained were always lower than 1 (DaII << 1).

A schematic representation of the Langmuir–Hinshelwood mechanism of 4-Nip reduction on the surface of Pd nanoparticles has been presented, and kinetic evaluation of the fitting of experimental data to the theoretical values has been done in the intrinsic kinetic area by using the Langmuir–Hinshelwood equation. The theoretical values obtained from the Langmuir–Hinshelwood equation were successfully fitted to the experimental data, and the true rate constant k, as well as the adsorption constants of 4-Nip and BH_4_^−^, were determined for each temperature.

The Arrhenius analysis of ln k of the inverse of the temperature led to the value of the true activation energy *Ea* (61 kJ·mol^−1^) of the true rate constant k. However, the thermodynamic parameters of the adsorption enthalpy ΔH and entropy ΔS for both reactants were determined since they depend on the temperature. Moreover, the thermodynamic parameters including the apparent activation energy (*Ea_app_*), pre-exponential factor A, activation of enthalpy ΔH^‡^, entropy ΔS^‡^, and Gibbs energy ΔG^‡^ were obtained by conducting the reaction at various temperatures and by using Arrhenius, Eyring, and Gibbs equations. The values of these thermodynamic parameters are in agreement of what is reported in the literature [19].

The results show that to compare the catalytic performance of mNPs, the reactions conditions should be the same such as using a small amount of mNPs, and the ratio of sodium borohydride to the 4-Nip concentration should be between 200 to 60 eq, so that apparent rate constant “*k_app_*” remains constant and is not strongly affected by these conditions parameters. Therefore, it appears that there is a crucial need for standardization of reaction conditions for a fair comparison of the catalytic performance of mNPs.

## Figures and Tables

**Table 1:** The result of thermodynamic parameters for 150 eq and 200 eq of BH_4_^−^ for Reduction of 4-Nip.

BH_4_^−^	*Ea_app_* (kJ·mol^−1^)	*A*	ΔH^‡^ (kJ·mol^−1^)	ΔS^‡^ (J·mol^−1^·K^−1^)	ΔG^‡^ (kJ·mol^−1^)
150 eq	81.22	1.46 × 10^11^	78.68	−39.57	90.47
200 eq	112.77	7.13 × 10^16^	110.22	69.36	89.56

**Table 2:** The summary of DaII Parameter Values for different amounts of Pd NPs at two NaBH_4_ equivalents.

*a* (m^−1^)	*β* (m/s)	*δ* (nm)	*k_app_*^a^ (×10^−3^ s^−1^)	DaII ^a^ (×10^−4^)	*k_app_*^b^ (×10^−3^ s^−1^)	DaII ^b^ (×10^−4^)
4613.1	0.077	9.045	34.05	0.965	10.23	0.290
1858.5	0.077	9.045	24.50	1.723	4.22	0.297
929.3	0.077	9.045	17.62	2.478	0.95	0.134
371.7	0.077	9.045	0.55	5.453	0.02	0.221

^a^ and ^b^ are 10,000 and 50 equivalents of NaBH_4_, respectively.

**Table 3:** The result of the rate constant k and the adsorption constants *K*_4-*Nip*_ and *K*_*BH*4−_ at different temperatures from fitting the experimental Data with the Langmuir–Hinshelwood model.

*T* (K)	*k* (mol/m^2^·s)	*K*_4-*Nip*_ (L/mol)	*K*_*BH*4−_ (L/mol)
317	0.02	8900	2.45
307	0.0115	6300	2.34
303	0.0083	5200	2.3
301	0.007	4800	2.28
300	0.006	4600	2.27
297.8	0.005	4200	2.25
296.3	0.0037	3900	2.235

**Table 4:** Summary of thermodynamic parameters for the adsorption process of 4-Nip and BH_4_^−^

	*K*_4-*Nip*_	*K*_*BH*4_^−^
ΔH_ad_ [kJ/mol]	30.87	3.31
ΔS_ad_ [J/mol K]	173	17.8

## Supplementary Materials

The following are available online at https://www.mdpi.com/2079-4991/10/6/1169/s1, The calculation of the surface area of the Pd nanoparticles, The general preparation of the Pd nanoparticles, The simulation of Langmuir-Hinshelwood equation with the MatLab routine used.
