# Supplementary material for: Kinetic Analysis of 4-Nitrophenol Reduction by “Water-Soluble” Palladium Nanoparticles
Source: Nanomaterials (Basel). 2020 Jun 15;10(6):1169. doi: 10.3390/nano10061169 (PMC7353196; doi:10.3390/nano10061169)
Supplement: Supplementary file 1 [file nanomaterials-10-01169-s001.pdf]

Calculating the surface area of the Pd nanoparticles:

The total surface area of the catalysts used in the catalytic reaction ($S_T\ (Pd)_{Nano}$) was calculated based on the number of Pd atoms per nanoparticles and median radius (r_{nano}) of the Pd nanoparticles.

Nanoparticles of Pd are considered as a compact sphere of radius r_{nano}. The surface area of total nanoparticles:

$$S_T\ (Pd)_{Nano} = S\ (Pd)_{Nano}\ (Pd)_{Nano}$$

With $S\ (Pd)_{Nano}$ the surface area of a *Pd* nano and $(Pd)_{Nano}$ The number of nanoparticles:

$$S\ (Pd)_{Nano} = 4\pi r_{nano}^2$$

$$(Pd)_{Nano} = \frac{(Pd)_{Atom}}{n(Pd)_{Atom}}$$

$n(Pd)_{Atom}$ are the number of Pd atoms per nanoparticles and $(Pd)_{Atom}$ are the total amount of Pd atoms for a solution of known concentration [Pd]:

$$(Pd)_{Atom} = [Pd] \times NA$$

With *NA* the Avogadro constant (NA = $6.02214076 \times 10^{23}$ mol^{-1})

Each crystalline lattice containing 4 Pd atoms, so the number of Pd atoms per nanoparticles:

$$n(Pd)_{Atom} = 4\frac{V_{sphere}}{V_{lattice}}$$

With $V_{lattice}$ the crystalline lattice volume:

$$n(Pd)_{Atom} = \frac{16\ \pi\ r_{nano}^3}{3\ V_{lattice}}$$

General preparation of the Pd nanoparticles:

In a 10 mL vial, 2 mL of distilled water, 80 µL of a water solution of Na_2PdCl_4 (20 mM) and 180 µL of bisphosphonic acid solution in water (40 mM HMBP aromatic) are mixed. Then 40 µL of a solution of sodium ascorbate (17.6 mg mL^{-1}) were added and the mixture was heated on a microwave apparatus (Monowave 300, Anton Paar GmbH, Graz, Austria) for 30 min at 80 °C using a ruby thermometer (step 1: heat as fast as possible to 100 °C and step 2: hold at 100 °C for 30 min with the stirring speed being 1200 rpm for both steps).

Simulation of Langmuir-Hinshelwood equation:

The concentration of 4-nitrophenol as the function of reaction time ($C_{4\text{-}Nip,exp}$) was simulated by a numerical solution of equation 1 by one Matlab routine. This Matlab routine was used to calculate the theoretical 4-nitrophenol concentration ($C_{4\text{-}Nip,ther}$) as the function of reaction time for given values of the true rate constant k and the Langmuir-Hinshelwood parameters $K_{4\text{-}Nip}$ and K_{BH4^-}. These values are changed and calculation was repeated until the agreement of the experimental concentration of 4-nitrophenol to theoretical values obtained from equation 1.

MatLab Routine:

```
function [t,X]=main
global X time y ;
v=load('Exp1.txt')
time=v(:,1);
y=v(:,2);
%K_4Nip0 =5800;
%K_BH40= 2.3;
k0 = 0.015;
M0=[ k0 ]
fminsearch(@param,M0);
end
%%%%%%%%%%%%%%%%%%%%%%%%%%%%%%%%%%%%
function [S] = param(Mp)
global K_4Nip K_BH4 k n time y
k = Mp(1);
%K_4Nip = Mp(2);
%K_BH4 = Mp(3);
% The initial C_4-Nip value (mol/L)
Xo = [7.38*10^-5];
[t,X] = ode45(@fun,time,Xo);
ysim=X(:,1);
S=0;
for j=1:length(y)
S=S+(y(j)-ysim(j))^2;
end
S
K_4Nipa=K_4Nip
K_BH4a=K_BH4
ka=k
na=n
figure(1)
%plot the results
plot(time,y,'--',t,X(:,1))
legend('C4-Nip,exp','C4-Nip,ther');
ylabel('C4-Nip(mol/L)');
xlabel('time(s)');
dlmwrite('sol.dat',[t,X],' ');
end
%%%%%%%%%%%%%%%%%%%%%%%%%%%%%%%%%%%%
function [dx_dt]= fun(t,x)
global K_4Nip K_BH4 k n
C_BH4=1.5*10^-2;
K_BH4=1.5;
```

```
a = K_BH4*C_BH4;
%k = 0.0053;
S=0.002453465;
n = 1;
b=4200;
K_4Nip=b
kS=k*S;
if x(1)>=0
dx_dt(1) =-((kS *(a)* (b* x(1))^n)/(1+ a +((b* x(1))^n))^2);
end
if x(1)<0
dx_dt(1)=0;
x(1)=0;
end
dx_dt = (dx_dt)';
end
```
